# Supplementary material for: Global burden of fall and associated factors among individual with low vision: A systematic-review and meta-analysis
Source: PLoS One. 2024 Jul 24;19(7):e0302428. doi: 10.1371/journal.pone.0302428 (PMC11268632; doi:10.1371/journal.pone.0302428)
Supplement: Attachment — Submitted filename: comments to low vision.docx [file pone.0302428.s003.docx]

Global Burden of fall and Associated factors among Individual with Low 2 Vision: A Systematic-Review and Meta-Analysis; It is an important piece of work but needs minor revision

Comments to author

- The title on the system and word document is different , should be similar

Global Burden of Fall Among Individual with Low Vision: A Systematic-Review and

Meta-Analysis and Global Burden of Fall and associated factors Among Individual with Low Vision: A Systematic-Review and

Meta-Analysis

- On page 1 line number 5 , Kingsley Ekemiri 1* 3 ,Chioma Ekemiri², Ngozika Ezinne¹,Victor Virginia³,Osaze 4 Okoendo⁴ , Robin Seemongal-Dass⁵ , Diane Van Staden⁶ , Carl Halladay Abraham⁷ , Low Vision Study Group¹

“ Low Vision Study Group” should be removed because it not the name of author

- The Corresponding Author is already showed by astrix and the affiliation so don’t need to be restated in title page
- Dr Kingsley Ekemiri(OD,MPH)
- Department of Optometry, Faculty of Medical Sciences, The University of West indies, St Augustine Campus, Trinidad and Tobago
- Under inclusion criteria- why authors only included reported fall injury within past two years?
- All included studies are published studies , why author dealt with unpublished study?
- Under conclusion- author recommended …to give attention, do you think there is no attention for those groups?
- The contents of declaration letter is not correct, it is cover letter , please amend it

See plos one submission guideline at

<https://journals.plos.org/plosone/s/submission-guidelines>

- All supporting information is not available
