# Supplementary material for: Global burden of fall and associated factors among individual with low vision: A systematic-review and meta-analysis
Source: PLoS One. 2024 Jul 24;19(7):e0302428. doi: 10.1371/journal.pone.0302428 (PMC11268632; doi:10.1371/journal.pone.0302428)
Supplement: Attachment — Submitted filename: PLOS ONE.docx [file pone.0302428.s004.docx]

manuscript PONE-D-23-44176

Global Burden of Fall Among Individual with Low Vision: A Systematic-Review and Meta-Analysis

Comments

The tittle “Global Burden of Fall among Individual with Low Vision: A Systematic-Review and Meta-Analysis” is important because, the Vision loss and fall are interrelated, vision loss is high among those who fall, and vision loss may be a contributing factor to falls. Falling results poor health and well-being, decreased activity of daily living and social participation, lower life satisfaction, as well as it affects the quality of life of the individuals.

I like to give minor comments and questions for this review.

1. I need to see the searching terms that you use for each data bases with their results? How do you get an access for each data bases other than PubMed?
2. The whole article has language and grammar problems.
3. In line 129 it says “the keywords used were, Low vision OR visual impairment OR…..” are this key words or search terms?
4. Check for the right citation: for example for citation 35………
5. Make the PRISMA diagram aesthetic.
6. Since you are using Preferred Reporting Items for Systematic Reviews and Meta-analyses (PRISMA)….. use its correct format. Like 1. Introduction, 2. Material and methods, 2.1. Study design and search strategy, 2.2. Study selection and eligibility criteria, 2.3. Study selection and quality appraisa, 2.4. Data extraction, 2.5. Measure, 2 .6. Statistical analysis, 2. 7. Publication bias, 3. Results………….. 4. Discussion, 4.1. Implications, 5. Conclusions, Financial disclosures, Declaration of Competing Interest, Acknowledgements, Appendix A. Supplementary data, References.
7. In line 235 make it 88.4%, it says 884%.

If the Egger’s test is found to be significant, it indicates the presence of publication bias in the meta-analysis. [To correct the final pooled prevalence, one can use the trim-and-fill method^1^](https://training.cochrane.org/resource/identifying-publication-bias-meta-analyses-continuous-outcomes). This method involves trimming the studies that are causing funnel plot asymmetry and then filling the funnel plot with hypothetical studies to make it symmetrical. The final pooled prevalence is then calculated using the filled funnel plot.
