# Supplementary material for: Global burden of fall and associated factors among individual with low vision: A systematic-review and meta-analysis
Source: PLoS One. 2024 Jul 24;19(7):e0302428. doi: 10.1371/journal.pone.0302428 (PMC11268632; doi:10.1371/journal.pone.0302428)
Supplement: Attachment — Submitted filename: response to reviewers (1).docx [file pone.0302428.s005.docx]

**Response for reviewer 1**

**Dear reviewer thank you for your constructive comments and suggestion, we tried to incorporate all issues raised by you.**

Manuscript PONE-D-23-44176 Global Burden of Fall Among Individual with Low Vision: A Systematic-Review and Meta-Analysis

Comments

The tittle “Global Burden of Fall among Individual with Low Vision: A Systematic-Review and Meta-Analysis” is important because, the Vision loss and fall are interrelated, vision loss is high among those who fall, and vision loss may be a contributing factor to falls. Falling results poor health and well-being, decreased activity of daily living and social participation, lower life satisfaction, as well as it affects the quality of life of the individuals.

I like to give minor comments and questions for this review.

1. I need to see the searching terms that you use for each data bases with their results? How do you get an access for each data bases other than PubMed?

**Response- as we mentioned in method part we used those search terms for all databases after we extracted MeSH for our study based on POCC.**

- **We got all most all studies from PubMed, from other sites we only get 2 studies ( 1 Scopus indexed, so we accessed through institutional login.) the other from Google scholar.**

**Regarding the access issue most of the health journals are indexed in PubMed in addition to that other sites, so we haven’t faced any challenges. But what we have done for those not indexed on those sited is we did manual searches.**

2. The whole article has language and grammar problems.

**Response- modified**

3. In line 129 it says “the keywords used were, Low vision OR visual impairment OR…..” are this key words or search terms?

**Response—corrected based on your suggestion**

4. Check for the right citation: for example for citation 35………

**Response—corrected**

5. Make the PRISMA diagram aesthetic.

**Response---corrected**

6. Since you are using Preferred Reporting Items for Systematic Reviews and Meta-analyses (PRISMA)….. use its correct format. Like 1. Introduction, 2. Material and methods, 2.1. Study design and search strategy, 2.2. Study selection and eligibility criteria, 2.3. Study selection and quality appraisa, 2.4. Data extraction, 2.5. Measure, 2 .6. Statistical analysis, 2. 7. Publication bias, 3. Results………….. 4. Discussion, 4.1. Implications, 5. Conclusions, Financial disclosures, Declaration of Competing Interest, Acknowledgements, Appendix A. Supplementary data, References.

**Response- we followed PRISMA 2020, so corrected based on your recommendation**

7. In line 235 make it 88.4%, it says 884%.

Response – corrected

8. If the Egger’s test is found to be significant, it indicates the presence of publication bias in the meta-analysis. To correct the final pooled prevalence, one can use the trim-and-fill method[^1^](https://training.cochrane.org/resource/identifying-publication-bias-meta-analyses-continuous-outcomes). This method involves trimming the studies that are causing funnel plot asymmetry and then filling the funnel plot with hypothetical studies to make it symmetrical. The final pooled prevalence is then calculated using the filled funnel plot.

Response – **mentioned on line 183 and 184**

**Response for reviewer 2**

**Dear reviewer thank you for your comments, we response for all comments raised by you.**

Global Burden of fall and Associated factors among Individual with Low 2 Vision: A Systematic-Review and Meta-Analysis; It is an important piece of work but needs minor revision

Comments to author

- The title on the system and word document is different , should be similar Global Burden of Fall Among Individual with Low Vision: A Systematic-Review and Meta-Analysis and Global Burden of Fall and associated factors Among Individual with Low Vision: A Systematic-Review and Meta-Analysis

**Response – corrected**

- On page 1 line number 5 , Kingsley Ekemiri 1* 3 ,Chioma Ekemiri², Ngozika

Ezinne¹,Victor Virginia³,Osaze 4 Okoendo⁴ , Robin Seemongal-Dass⁵ , Diane Van

Staden⁶ , Carl Halladay Abraham⁷ , Low Vision Study Group¹

“ Low Vision Study Group” should be removed because it not the name of author

**Response – removed**

- The Corresponding Author is already showed by astrix and the affiliation so don’t need to be restated in title page

Dr Kingsley Ekemiri(OD,MPH)

Department of Optometry, Faculty of Medical Sciences, The University of West indies, St Augustine Campus, Trinidad and Tobago

**Response – removed**

- Under inclusion criteria- why authors only included reported fall injury within past two years?

**Response – the author included studies which reported within two years is in order to minimize recall bias.**

- All included studies are published studies, why author dealt with unpublished study?

**Response – corrected and we removed the word unpublished from main document, we don’t have unpublished studies**

- Under conclusion- author recommended …to give attention, do you think there is no attention for those groups?

**Response – the current facts says fall is not given attention for those with low vision rather most literature suggests attention for older age groups**

- The contents of declaration letter is not correct, it is cover letter , please amend it

See plos one submission guideline at <https://journals.plos.org/plosone/s/submission-guidelines>

**Response – corrected and removed from the manuscript**

- All supporting information is not available

**Response – we will make available/ attach as supporting information**
